# Association of Ghrelin Gene Polymorphisms and Serum Ghrelin Levels with the Risk of Hepatitis B Virus-Related Liver Diseases in a Chinese Population

**DOI:** 10.1371/journal.pone.0143069

**Published:** 2015-11-23

**Authors:** Xiaolian Zhang, Limin Zhai, Chengzhi Rong, Xue Qin, Shan Li

**Affiliations:** Department of Clinical Laboratory, First Affiliated Hospital of Guangxi Medical University, Nanning, Guangxi, China; National Health Research Institutes, TAIWAN

## Abstract

**Background:**

The functions of ghrelin (GHRL) include anti-inflammatory effects, reduction of the fibrogenic response, protection of liver tissue, and regulation of cell proliferation. Genetic variations in the GHRL gene may play an important role in the development of chronic hepatitis B (CHB), liver cirrhosis (LC) and hepatocellular carcinoma (HCC). Therefore, we investigated whether GHRL gene polymorphisms and its serum levels are associated with hepatitis B virus (HBV)-related diseases risk in a Chinese population.

**Methods:**

176 patients with CHB, 106 patients with HBV-related LC, 151 patients with HBV-related HCC, and 167 healthy controls were recruited in the study. Genotyping of GHRL rs26311, rs27647, rs696217, and rs34911341 polymorphisms were determined with the polymerase chain reaction–restriction fragment length polymorphism (PCR–RFLP) and DNA sequencing. The serum GHRL concentrations were determined using enzyme-linked immunosorbent assay (ELISA).

**Results:**

Binary logistic regression analyses adjusting for gender and age revealed that a significant increased risk of LC was found in the GHRL rs26311 GC genotype and combined GC+CC genotypes when compared with the GG genotype (GC vs. GG: OR = 1.671, 95% CI = 1.013–2.757, *P* = 0.044; GC+CC vs. GG: OR = 1.674, 95% CI = 1.040–2.696, *P* = 0.034). In subgroup analysis by gender, binary logistic regression analyses adjusting for age showed that the GHRL rs26311 C allele and combined GC+CC genotypes were associated with a significantly increased risk to LC in males (C vs. G OR = 1.416, 95% CI = 1.017–1.972, *P* = 0.040; GC+CC vs. GG: OR = 1.729, 95% CI = 1.019–2.933, *P* = 0.042). In addition, we found significant decreased serum GHRL levels in LC patients compared with the healthy controls. However, there was no significant association of the GHRL rs26311 polymorphism with serum GHRL levels in LC patients.

**Conclusions:**

These observations suggest that the GHRL rs26311 polymorphism is associated with an increased risk to HBV-related LC, especially in men. We also found an inverse association of serum GHRL levels with LC.

## Introduction

The consequences of acute and chronic hepatitis B virus (HBV) infections remain a major public health problem globally, especially in Sub-Saharan Africa and most of Asia [1]. Two billion people have been infected with HBV, and tens of millions additional infections occur annually world-wide [2]. Of those who carry HBV, 350 to 400 million people persistently infected with HBV develop chronic hepatitis, which leads to approximately one-third of all cases of liver cirrhosis (LC) and more than three quarters of all cases of hepatocellular carcinoma (HCC) [3, 4]. It is widely accepted that HBV induces inflammation of the liver, mediated by cytotoxic T lymphocytes against infected hepatocytes, leading to oxidative stress and hepatic cell death and regeneration [5, 6]. Chronic liver inflammation can lead to chronic hepatitis B (CHB) and LC and eventually increase susceptibility to HCC [7].

CHB, LC, and HCC are progressive stages of chronic HBV infection [8]. Apart from immunological factors, viral factors, environmental factors, and host genetic factors may contribute to HBV progression [9]. Ghrelin (GHRL) is a 28 amino acid gastrointestinal peptide hormone that was first isolated in human and rat stomachs as a novel endogenous ligand for the growth hormone secretagogue receptor [10, 11]. GHRL has various metabolic functions, including regulation of energy homeostasis, stimulation of gastric acid secretion, and regulation of gastrointestinal motility [12, 13]. Other effects include diverse cardiovascular functions, reproductive functions, and anti-inflammatory actions [14–16]. GHRL also reduces the fibrogenic response and decreases liver injury and hepatoprotective effects on chronically injured tissues [17, 18]. Moreover, GHRL has been reported to regulate processes associated with cancer, including cell proliferation, apoptosis, cell migration, cell invasion, inflammation, and angiogenesis [19, 20].

The gene encoding GHRL is located on chromosome 3p26-25 and contains four exons and three introns [21]. There are at least 263 single nucleotide polymorphisms (SNPs) in GHRL, reported in the dbSNP database (http://www.ncbi.nlm.nih.gov/snp/). Four SNPs of the GHRL gene (rs26311, rs27647, rs696217, and rs34911341) have been extensively studied [22, 23]. GHRL SNPs rs26311 and rs27647 are the two common coding SNPs in the promoter region. The GHRL rs696217 polymorphism is characterized by a G to T substitution resulting in a leucine (Leu) to methionine (Met) exchange at position 72 in exon 2 of the coding region of the preproghrelin gene, whereas the rs34911341 polymorphism is characterized by a C to T transition causing an arginine (Arg) to glutamine (Gln) exchange at position 51 in exon 2. In addition, these genetic variations in the GHRL gene may affect the expression and function of GHRL protein, and thus modulate disease risk [24–26]. Hence, it is biologically reasonable to hypothesize a potential association between the GHRL rs26311, rs27647, rs696217, and rs34911341 polymorphisms with HBV-related diseases risk.

Several studies have investigated the association between the GHRL rs26311, rs27647, rs696217, and rs34911341 polymorphisms with the risk of various diseases such as obesity [25], metabolic syndrome [27, 28], type 2 diabetes [29], breast cancer [30], and colorectal cancer [31]. Until now, there were only two studies that examined the association between GHRL polymorphisms and the risk of liver diseases [17, 26]. One study assessing 284 Spanish patients with chronic hepatitis C suggested that GHRL rs26312 and rs27647 polymorphisms influenced the progression of liver fibrosis in patients with this illness [17]. Another study including 40 Egyptian patients with chronic hepatitis C, 39 patients with hepatitis C virus (HCV)-related HCC, and 40 healthy subjects reported that patients with HCV-related HCC carrying GHRL rs34911341 A allele had a significantly higher susceptibility to developing HCC when compared with controls [26].

In addition, the genotype and allele distributions of GHRL polymorphisms vary with race and ethnicity, but to date no data have been available concerning the association of GHRL polymorphisms with HBV-related diseases in a Chinese population. The correlation between GHRL gene polymorphisms and serum GHRL levels remains unknown. Therefore, this study aimed to investigate whether SNPs rs26311, rs27647, rs696217, and rs34911341 of the GHRL gene and serum GHRL levels are associated with the risk to Chinese patients with HBV-related liver diseases.

## Materials and Methods

### Study population

This was a hospital-based case-control study of 600 subjects, including 176 patients with CHB (149 males, 27 females, mean age 39.25 years), 106 patients with HBV-related LC (88 males, 18 females, mean age 46.82 years), 151 patients with HBV-related HCC (131 males, 20 females, mean age 49.32 years), and 167 healthy controls (144 males, 23 females, mean age 37.78 years). All patients with HBV-infected diseases were unrelated and consecutively recruited for this study at the First Affiliated Hospital of Guangxi Medical University (Guangxi, China) from 1st June to 1st December of 2013. All patients were positive for HBV surface antigen (HBsAg), HBV core antibody (HbcAb), hepatitis B e antigen (HBeAg) or hepatitis B e antibody (HBeAb) for a period of at least 6 months. CHB was further diagnosed by serum HBV-DNA level ≥1000 IU/mL. HBV-related LC was defined by pathologic examination or typical morphologic findings from computed tomography (CT) or ultrasonography as well as laboratory features. The diagnosis of HBV-related HCC was based on alpha-fetoprotein (AFP) levels >400 ng/mL and/or pathological confirmation; and/or positive finding on computed tomography (CT), magnetic resonance imaging (MRI) or ultrasonography combined.

The healthy participants who were matched to cases on the basis of gender and age underwent a general health check-up at the Department Medical Centre of the First Affiliated Hospital of Guangxi Medical University during the same time frame. The selection criteria for control subjects were that patients could have no clinical evidence of cancer or other serious illness or a family history of cancer or other serious illness. All subjects provided written informed consent after an explanation of the study. The study protocol was approved by the ethics committee of the First Affiliated Hospital of Guangxi Medical University (Guangxi, China) and the methods were carried out in accordance with the approved guidelines.

### DNA extraction and SNP genotyping

Two milliliters of venous whole blood were drawn from each subject and collected in tubes with EDTA-K_2_ as the anticoagulant. The tubes were stored at -80°C until DNA extraction. Genomic DNA was isolated from venous blood using the standard phenol–chloroform method. Genotypes of GHRL SNPs rs26311, rs27647, rs696217 and rs34911341 were determined with polymerase chain reaction–restriction fragment length polymorphism (PCR–RFLP). Primer sequences, annealing temperature, restriction enzymes and product size in SNPs genotyping are shown in Table 1.

**Table 1:** Primer sequence and the reaction condition for genotyping GHRL polymorphisms.

SNPs	Primer sequence (5'→3')	Annealing temperature	Restriction enzyme	Product size (bp)
GHRL rs26311	F: GCGTAGATCTTCCACCTCCA	55°C	BcnI	GG: 228+61
(-1062G>C)	R: CGTTGTTTCCCATGTGCTGT		37°C for 6h	GC: 289+228+61
				CC: 289
GHRL rs27647	F: CACAGCAACAAAGCTGCACC	60°C	Dra-I	TT: 664+265
(-604A/G)	R: AAGTCCAGCCAGAGCATGCC		37°C for 6h	TC: 929+664+265
				CC: 929
GHRL rs696217	F: GCTGGGCTCCTACCTGAGC	60°C	Bsr-I	GG: 517+101
(408C/A)	R: GGACCCTGTTCACTGCCAC		65°C for 3h	GT: 618+517+101
				TT: 618
GHRL rs34911341	F: GCTGGGCTCCTACCTGAGC	60°C	Sac-I	CC: 455+163
(346G/A)	R: GGACCCTGTTCACTGCCAC		37°C for 6h	CT: 618+455+163
				TT: 618

The PCR amplification was conducted in a final 25 μL reaction mixture comprising 2 μL of genomic DNA (60 ng/μL), 1 μL of forward primer (10 μmol/L), 1 μL of reverse primer (10 μmol/L), 12.5 μL of DreamTaq Green PCR Master Mix (Thermo Fisher Scientific), and 8.5 μL of nuclease-free deionized water using a Perkin-Elmer thermocycler (2700, Applied Biosystems, Foster City, CA, USA). For GHRL SNPs rs26311, rs27647, rs696217 and rs34911341 10 μL of the PCR product was added to 1 μL of BcnI, Dra-I, Bsr-I, and Sac-I restriction endonuclease (Thermo Fisher Scientific), respectively.

**Fig 1:**
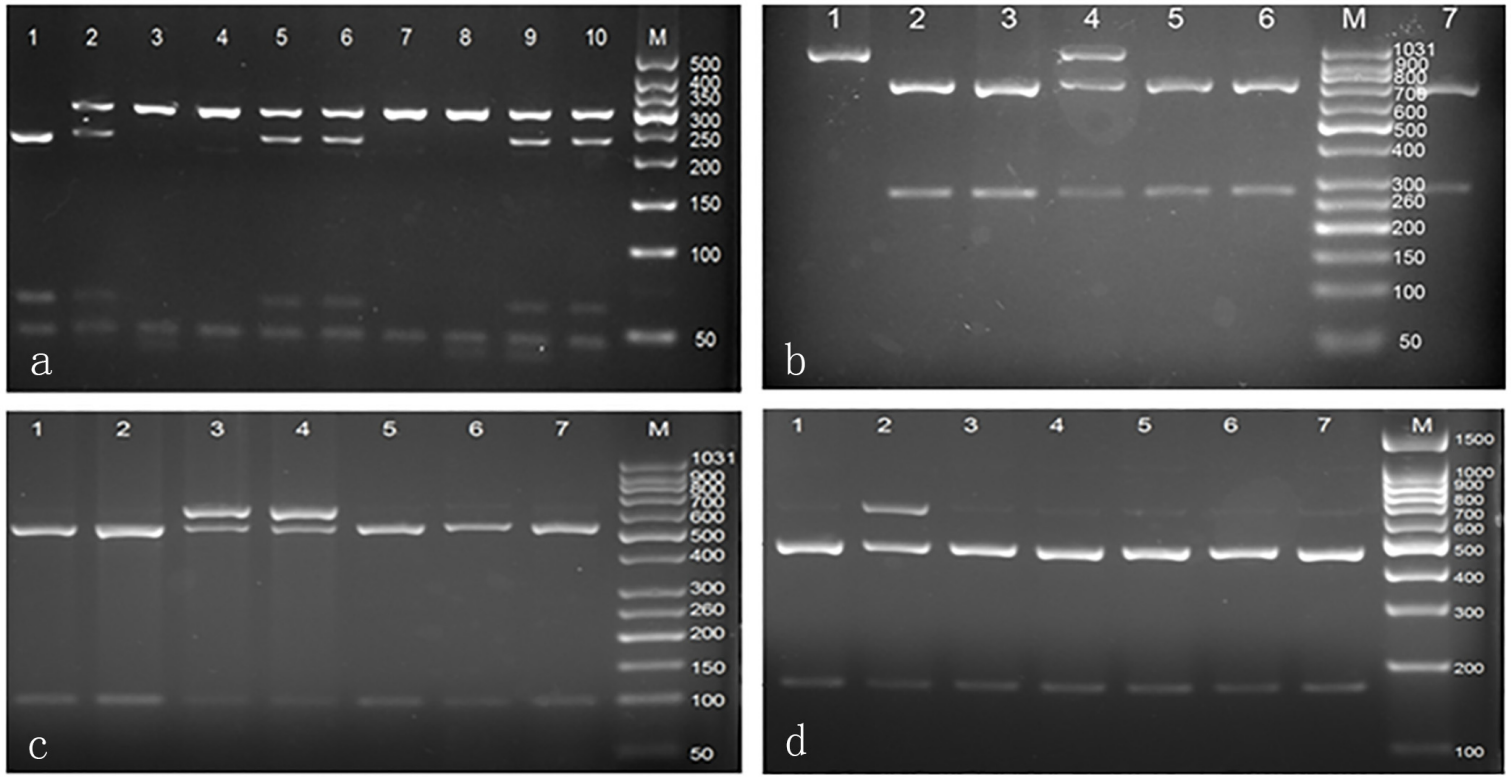
PCR-RFLP assay for analyzing the rs26311, rs27647, rs696217, and rs34911341 polymorphisms in GHRL gene. (a) rs26311—lane M shows DNA marker; lane 1 shows GG genotype; lanes 2, 5, 6, 9, and 10 show GC genotype; lanes 3, 4, 7, and 8 show CC genotype. (b) rs27647—lane M shows DNA marker; lanes 2, 3, 5, 6, and 7 show TT genotype; lane 4 shows TC genotype; lane 1 shows CC genotype. (c) rs696217—lane M shows DNA marker; lanes 1, 2, 5, 6, and 7 show GG genotype; lanes 3 and 4 show GT genotype. (d) rs34911341—lane M shows DNA marker; lanes 1, 3, 4, 5, 6, and 7 show CC genotype; lane 2 show CT genotype.

During the next step, the digested DNA fragments were separated on a 1.5% agarose gel and visualised under ultraviolet light after staining with GoldView I (Thermo Fisher Scientific) (Fig 1). Band size was determined using a DNA marker (CWBIO, Beijing, China). To confirm the genotyping results, 10% of the individual specimens in each group (60 samples) were randomly chosen for genotyping by DNA sequencing using an ABI Prism 3100 (Applied Biosystems, Shanghai Sangon Biological Engineering Technology & Services Co., Ltd., Shanghai, China). The results of DNA sequencing and PCR–RFLP were 100% consistent.

### Serum GHRL measurement

About four milliliters of venous whole blood was obtained from each participant after an overnight fast of 12 h and placed into serum tubes (Becton, Dickinson and Company, Franklin Lakes, New Jersey, USA). Blood samples were allowed to clot for 30 min at 4°C and then centrifuged at 3000 rpm for 10 min. The serum was separated and stored at -80°C for determination of fasting GHRL concentrations. Total serum GHRL level was evaluated using a human growth hormone releasing peptide-GHRL enzyme-linked immunosorbent assay (ELISA) Kit (Sangon Biotech, Shanghai, China) with an assay range of 5 to 160 μg/L according to the manufacturer’s instructions. An ELISA reader (680, BIO-RAD, California, USA) was used to read absorbance at 450nm to analyze serum GHRL levels.

### Statistical analysis

Normality was tested by Shapiro-Wilk test and D’Agostino test. Normally distributed variables were shown as means and standard deviations (SDs), non-normally distributed variables were presented as medians and interquartile ranges (IQRs). If data distribution is normal, difference among groups were compared by parametric test, otherwise non-parametric test was applied. Demographic characteristics and clinical parameters among the groups were evaluated with a χ^2^ test for categorical variables and a one-way analysis of variance (ANOVA) test for continuous variables. Subsequent pair-wise comparisons were carried out by least significance difference *t* (LSD-*t*) and Student-Newman-Keuls (SNK) tests. Genotype distributions in all studied groups for each SNP were tested for Hardy-Weinberg equilibrium (HWE) using the GenePop web program (http://ihg.gsf.de/cgi-bin/hw/hwa1.pl). Genotype and allele frequencies of GHRL polymorphisms were compared among different groups using the χ^2^ test and Fischer’s exact test. Binary logistic regression was used to calculate odds ratios (ORs) and 95% confidence intervals (CIs) after adjusting for gender and age to evaluate the relative risk conferred by a particular allele and genotype. A stratified analysis based on gender was performed to explore gender’s effect on the association between GHRL polymorphisms and risk of HBV-related diseases. Haplotype analyses and linkage disequilibrium were performed using the SHEsis software [32]. The statistical software SPSS version 16.0 (SPSS Inc, Chicago, IL, USA) was used for data analysis. A 2-tailed *P* value of <0.05 was considered statistically significant.

## Results

### Characteristics of the study population

The demographic data and clinical parameters of the controls and case subjects enrolled in this study are shown in Table 2. The demographic characteristics included gender and age; the laboratory features included alanine aminotransferase (ALT), aspartate aminotransferase (AST) and AFP. No significant difference existed between the cases and controls for the gender distribution (*P* = 0.883). The healthy controls and CHB cases were significantly younger than the LC cases and HCC cases (*P*<0.001). The data related to the clinical parameters showed that serum ALT and AST levels were significantly increased in CHB patients when compared to the healthy controls, LC patients, and HCC patients (*P*<0.001). Moreover, compared with the control group and the groups with CHB and LC, the group with HCC had significantly higher AFP concentrations (*P*<0.001).

**Table 2:** Demographic and laboratory characteristics of all subjects. CHB, chronic hepatitis B; LC, liver cirrhosis; HCC, hepatocellular carcinoma; SD, standard deviation; ALT, alanine aminotransferase; AST, aspartate aminotransferase; AFP, alpha-fetoprotein.

Variables	Controls	CHB	LC	HCC	*P*
Overall	167	176	106	151	
Demographic parameters					
Gender (M/F)	144/23	149/27	88/18	131/20	0.833
Age (years, mean±SD)	37.78±11.83	39.25±11.49	46.82±9.15	49.32±11.29	<0.001
Laboratory parameters (mean±SD)					
ALT (IU/L)	23.64±11.15	233.40±296.68	72.11±84.84	53.57±47.25	<0.001
AST (IU/L)	21.31±4.71	172.29±240.50	107.73±102.48	62.09±61.02	<0.001
AFP (ng/mL)	3.09±1.53	65.83±90.90	68.08±106.86	429.43±522.97	<0.001

### Association of GHRL gene polymorphisms with the risk of HBV-related diseases

Among the 600 subjects, only one subject (among the control subjects) had the CT genotype for the GHRL rs34911341 polymorphism; the rest were wild-type CC homozygote. The genotype and allele frequencies of the GHRL rs26311, rs27647, and rs696217 polymorphisms between patients with HBV-related diseases and healthy controls are listed in Table 3. The genotype distributions of rs26311, rs27647 and rs696217 were in accordance with HWE in all studied groups (Table 3).

**Table 3:** Association analysis of GHRL polymorphisms between HBV-related patients and healthy controls. HBV, hepatitis B virus; SNPs, single nucleotide polymorphisms; CHB, chronic hepatitis B; LC, liver cirrhosis; HCC, hepatocellular carcinoma; OR, odds ratio; CI, confidence interval; HWE, Hardy–Weinberg equilibrium.

	Controls	CHB			LC			HCC		
SNPs	N = 167 (%)	N = 176 (%)	OR (95% CI)	*P*	N = 106 (%)	OR (95% CI)	*P*	N = 151 (%)	OR (95% CI)	*P*
rs26311										
GG	62 (37.1)	65 (36.9)	1		29 (27.4)	1		63 (41.7)	1	
GC	76 (45.5)	80 (45.5)	0.991 (0.667–1.471)	0.964	53 (50.0)	**1.671 (1.013–2.757)**	**0.044**	68 (45.0)	0.996 (0.652–1.552)	0.986
CC	29 (17.4)	31 (17.6)	1.036 (0.626–1.713)	0.892	24 (22.6)	1.683 (0.925–3.061)	0.088	20 (13.3)	0.644 (0.365–1.138)	0.130
GC+CC	105 (62.9)	111 (61.4)	1.002 (0.689–1.458)	0.991	77 (72.6)	**1.674 (1.040–2.696)**	**0.034**	88 (58.3)	0.895 (0.599–1.337)	0.587
G alleles	200 (59.9)	210 (59.7)	1		111 (52.4)	1		194 (64.2)	1	
C alleles	134 (40.1)	142 (40.3)	1.017 (0.785–1.317)	0.900	101 (47.6)	1.343 (0.995–1.813)	0.054	108 (35.8)	0.845 (0.641–1.115)	0.233
*P* ^-HWE^	0.495	0.460			0.982			0.807		
rs27647										
TT	132 (79.0)	139 (79.0)	1		79 (74.5)	1		119 (78.8)	1	
TC	34 (20.4)	34 (19.3)	0.962 (0.664–1.329)	0.837	27 (25.5)	1.031 (0.659–1.613)	0.859	32 (21.2)	0.878 (0.593–1.301)	0.517
CC	1 (0.6)	3 (1.7)	3.287 (0.631–17.115)	0.158	0 (0.0)	-	-	0 (0.0)	-	-
TC+CC	35 (21.0)	37 (21.0)	0.969 (0.691–1.361)	0.856	27 (25.5)	1.031 (0.659–1.613)	0.859	32 (21.2)	0.878 (0.593–1.301)	0.517
T alleles	298 (89.2)	312 (88.6)	1		185 (87.3)	1		270 (89.4)	1	
C alleles	36 (10.8)	40 (11.4)	1.028 (0.777–1.360)	0.848	27 (12.7)	1.227 (0.902–1.669)	0.193	32 (10.6)	0.869 (0.645–1.171)	0.356
*P* ^-HWE^	0.449	0.586			0.133			0.145		
rs696217										
GG	112 (67.1)	111 (63.1)	1		65 (61.3)	1		102 (67.6)	1	
GT	50 (29.9)	60 (34.1)	1.264 (0.895–1.785)	0.183	37 (34.9)	1.349 (0.905–2.011)	0.141	42 (27.8)	0.891 (0.614–1.293)	0.544
TT	5 (3.0)	5 (2.8)	1.199 (0.474–3.030)	0.702	4 (3.8)	1.203 (0.445–3.252)	0.716	7 (4.6)	1.417 (0.579–3.469)	0.445
GT+TT	55 (32.9)	65 (36.9)	0.798 (0.578–1.102)	0.170	41 (38.7)	0.749 (0.516–1.088)	0.129	49 (32.4)	1.067 (0.757–1.503)	0.711
G alleles	274 (82.0)	282 (80.1)	1		167 (78.8)	1		246 (81.5)	1	
T alleles	60 (18.0)	70 (19.9)	1.219 (0.903–1.647)	0.196	45 (21.2)	1.246 (0.888–1.748)	0.203	56 (18.5)	1.004 (0.729–1.383)	0.980
*P* ^-HWE^	0.838	0.354			0.652			0.330		

In the genotype analysis, no significant differences existed in the genotype distributions of rs26311, rs27647 and rs696217 among groups (*P* = 0.286, *P* = 0.418, and *P* = 0.803, respectively), nor were there significant differences in the distributions of allele frequency among groups (*P* = 0.062, *P* = 0.879, and *P* = 0.783, respectively). With respect to the GHRL rs26311 polymorphism, binary logistic regression analyses adjusted by gender and age revealed that subjects carrying the rs26311 GC genotype (but not the CC genotype) experienced an increased risk of LC compared with those carrying the GG genotype (OR = 1.671, 95% CI = 1.013–2.757, *P* = 0.044). Similarly, a significant increased risk of LC was found in dominant model GC+CC vs. GG (OR = 1.674, 95% CI = 1.040–2.696, *P* = 0.034). Furthermore, the rs26311 polymorphism was not associated with CHB and HCC risk in any analytic model. Binary logistic regression analyses adjusted by gender and age failed to reveal any significant difference between GHRL rs27647 polymorphism with CHB, LC, and HCC risk. Similar non-significant results were also observed with respect to the GHRL rs696217 polymorphism.

### HBV-related LC patients compare with CHB patients

To elucidate and distinguish the effects of GHRL rs26311 polymorphism on HBV-related LC through direct influence on LC progression or indirect influence as induced by HBV, CHB patients were also selected as a control group, and binary logistic regression was used to estimate ORs and 95% CIs after adjusting for gender and age to test the relationship between the rs26311 polymorphism and the risk of LC compared with CHB patients. The results indicated that there was no significant difference between rs26311 polymorphism and LC risk when compared with CHB patients (C vs. G: OR = 0.781, 95% CI = 0.544–1.120, *P* = 0.178; GC vs. GG: OR = 0.721, 95% CI = 0.401–1.296, *P* = 0.275; CC vs. GG: OR = 1.144, 95% CI = 0.588–2.226, *P* = 0.691; GC+CC vs. GG: OR = 0.693, 95% CI = 0.399–1.202, *P* = 0.192).

### Association of GHRL gene polymorphisms with the risk of HBV-related diseases according to gender

In our stratified analysis of GHRL polymorphisms and risk of HBV-related diseases based on gender, no significant differences in the genotype and allele frequencies of rs26311, rs27647, and rs696217 among groups were observed. In the binary logistic regression analyses adjusted by age, we found that males who carried the rs26311 C allele were correlated with an increased risk of LC compared to the G allele (OR = 1.416, 95% CI = 1.017–1.972, *P* = 0.040). Similarly, male subjects carrying the rs26311 combined GC+CC genotypes were at increased risk of LC (OR = 1.729, 95% CI = 1.019–2.933, *P* = 0.042) (S1 Table). On the other hand, no significant differences were observed in females (S2 Table). With regard to GHRL rs27647 and rs696217 polymorphisms, binary logistic regression analyses did not reveal any significant differences in the genotype and allele distributions among groups.

### Comparison of genotypes distributions with the HapMap project data

Given that the GHRL genetic background may be distinct in different populations, the genotype and allele frequencies of the four SNPs in our control group were further compared to those different ethnicities’ healthy controls from the Haplotype Map (HapMap) project (http://www.ncbi.nlm.nih.gov/snp/). There was lack of HapMap data from HCB (Han Chinese in Beijing), JPT (Japanese in Tokyo), CEU (Utah residents with northern and western European ancestry) and YRI (Yoruba in Ibadan) populations of the SNP rs34911341. Thus, we only compared the genotype and allele frequencies of controls for rs26311, rs27647, and rs696217 polymorphisms with the above populations from the HapMap database. The data list in Table 4 indicates that the distributions of rs26311, rs27647, and rs696217 in our control group were similar to those in the HCB and JPT populations, but significantly different from those in CEU and YRI populations. For the rs26311 and rs696217 polymorphisms, the frequencies of genotype GG and allele G in CEU and YRI populations are significantly higher than frequencies in the healthy controls in the present study. At the rs27647 site, there is a significantly higher detection rate of the TT genotype and T allele in our data comparison with CEU and YRI populations.

**Table 4:** Comparison of genotype and allele frequencies in the healthy controls of the present study and that from the HapMap project. SNPs, single nucleotide polymorphisms; HCB, Han Chinese in Beijing, China; JPT, Japanese in Tokyo, Japan; CEU, Utah residents with northern and western Europeanancestry; YRI, Yoruba in Ibadan, Nigeria.

SNPs	Samples, N	Genotype frequency, n (%)			*P*	Allele frequency, n (%)		*P*
rs26311		GG	GC	CC		G	C	
Present Study	167	62 (37.1)	76 (45.5)	29 (17.4)		200 (59.9)	134 (40.1)	
HCB	45	19 (42.2)	21 (46.7)	5 (11.1)	0.572	59 (65.6)	31 (34.4)	0.394
JPT	45	19 (42.2)	21 (46.7)	5(11.1)	0.572	59 (65.6)	31 (34.4)	0.394
CEU	60	44 (73.3)	14 (23.3)	2 (3.3)	0.000	102 (85.0)	18 (15.0)	0.000
YRI	60	37 (61.7)	21 (35.0)	2 (3.3)	0.001	95 (79.2)	25 (20.8)	0.000
rs27647		TT	TC	CC		T	C	
Present Study	167	132 (79.0)	34 (20.4)	1 (0.6)		298 (89.2)	36 (10.8)	
HCB	43	33 (76.7)	8 (18.6)	2 (4.7)	0.135	74 (86.0)	12 (14.0)	0.447
JPT	86	68 (79.1)	18 (20.9)	0 (0)	0.770	154 (89.5)	18 (10.5)	0.914
CEU	113	49 (43.4)	51 (45.1)	13 (11.5)	0.000	149 (65.9)	77 (34.1)	0.000
YRI	113	62 (54.9)	39 (34.5)	12 (10.6)	0.000	163 (72.1)	63 (27.9)	0.000
rs696217		GG	GT	TT		G	T	
Present Study	167	112 (67.1)	50 (29.9)	5 (3.0)		274 (82.0)	60 (18.0)	
HCB	43	31 (72.1)	10 (23.3)	2 (4.7)	0.624	72 (83.7)	14 (16.3)	0.874
JPT	86	55 (64.0)	27 (31.4)	4 (4.7)	0.755	137 (79.7)	35 (20.3)	0.549
CEU	113	95 (84.1)	15 (13.3)	3 (2.7)	0.005	205 (90.7)	21 (9.3)	0.005
YRI	113	111 (98.2)	2 (1.8)	0 (0.0)	0.000	224 (99.1)	2 (0.9)	0.000

### Haplotype analysis of the GHRL gene polymorphisms and LC risk

Haplotype analyses were performed in the patients with LC and healthy controls using SHEsis software, and seven possible haplotype frequencies of GHRL rs26311, rs27647, and rs696217 are presented in Table 5. Overall, linkage disequilibrium was not obviously observed between the alleles. The GTG haplotype is the most common haplotype and accounted for 41.6% in healthy controls, whereas the haplotype with the highest frequency in the LC group was CTG, which accounted for 35.8% of the total among the seven haplotypes. Nevertheless, there were no significant differences in haplotype frequencies between the LC patients and healthy controls.

**Table 5:** Haplotype distribution in LC patients and healthy controls. LC, liver cirrhosis; OR, odds ratio; CI, confidence interval.

Haplotype	Controls	LC	OR (95% CI)	*P*
CCT	0.020	0.027	1.375 (0.444–4.254)	0.579
CTG	0.328	0.358	1.143 (0.796–1.641)	0.470
CTT	0.053	0.091	1.786 (0.915–3.488)	0.086
GCG	0.077	0.080	1.046 (0.551–1.983)	0.891
GCT	0.014	0.020	1.442 (0.388–5.362)	0.583
GTG	0.416	0.350	0.756 (0.529–1.080)	0.124
GTT	0.092	0.074	0.781 (0.414–1.473)	0.444

### Serum GHRL levels

Serum samples were available for 45 controls and 45 LC cases. GHRL levels of control and LC groups were abnormally distributed and they were reported as median and IQR (Table 6). Mann-Whitney *U* test showed decreased serum GHRL in LC patients (17.54±15.59 μg/L) compared with the healthy controls (48.84±33.32 μg/L, *P*<0.001). Similar significant results were also found when compared difference of serum GHRL values in two groups among the individuals with the same genotype of the rs26311 polymorphism. However, when considering the possible difference of GHRL levels among three different genotypes in the same group, no significant differences were observed, indicating that serum GHRL levels were not associated with the rs26311 polymorphism. Details are shown in Table 6.

**Table 6:** Association of GHRL rs26311 polymorphism with serum GHRL levels (median±IQR, μg/L) in LC patients and healthy controls. GHRL, ghrelin; LC, liver cirrhosis; IQR, interquartile range;

	Overall		rs26311 genotype						
Groups			GG		GC		CC		
	N	GHRL levels	N	GHRL levels	N	GHRL levels	N	GHRL levels	*P* *
Controls	45	48.84±33.32	12	55.25±34.86	22	42.84±41.43	11	51.38±18.43	0.803
LC	45	17.54±15.59	11	20.73±20.69	24	16.75±12.89	10	18.52±16.60	0.631
*P* **		0.000		0.011		0.000		0.000	

*Kruskal-Wallis *H* test, comparing the difference of serum GHRL levels among three different genotypes in the same group

**Mann-Whitney *U* test, comparing the difference of serum GHRL levels in two groups among the individuals with the same genotype.

## Discussion

In China, 120 million people are HBV carriers; 20 million suffer from CHB, and almost 300,000 die annually from chronic consequences of HBV infection, such as HBV-related LC or HCC [33]. It is well known that CHB, LC, and HCC are progressive stages of chronic HBV infection for which the underlying mechanisms are not well understood [8]. In the present study, we performed a large case-control study in 600 Chinese subjects to investigate whether four SNPs in the GHRL gene are associated with the presence of CHB, HBV-related LC, and HBV-related HCC. To the best of our knowledge, this is the first study conducted on the association between GHRL polymorphisms and the risk to HBV-related diseases. The results revealed that the GHRL rs26311 polymorphism was significantly associated with HBV-related LC. The rs26311 GC genotype and combined GC+CC genotypes were correlated with a significant increase in LC risk when compared with the wild-type GG homozygote. In stratified analysis by gender, the rs26311 C allele and combined GC+CC genotypes were observed to be significantly associated with LC in males. However, in haplotype analyses, we did not obtain any significant relationship among GHRL gene haplotypes and LC susceptibility. Our data show that the GHRL rs26311 polymorphism may contribute to increased risk of HBV-related LC in the Chinese population, particularly in males.

GHRL, a gastric mucosa-derived peptide, exerts a wide variety of physiological actions and influences, including growth hormone secretion, energy balance and appetite, gastric acid secretion and gastrointestinal motility, glucose homeostasis, and general influence on cardiovascular and reproductive functions [12, 13]. Moreover, subsequent in vitro and in vivo data have clearly shown the anti-inflammatory functions of GHRL. In vitro, GHRL has been demonstrated to inhibit the expression of pro-inflammatory cytokines, including IL-1β, IL-6 and TNF-α by human peripheral blood mononuclear cells (PBMCs) and T cells [16], and to reduce angiotensin II-induced (Ang II-induced) production of TNF-α, MCP-1, and IL-8 in human umbilical vein endothelial cells (HUVECs) [34]. In vivo, administration of ghrelin down-regulated endotoxin-induced pro-inflammatory cytokine release [35] and attenuated sepsis-induced acute lung injury [36], biliary obstruction-induced chronic hepatic injury [37], and non-alcoholic fatty liver disease (NAFLD)-induced liver injury and inflammation [38]. Therefore, it is reasonable to hypothesise that GHRL might correlate with inflammation-related diseases progression. The GHRL gene is located in chromosome 3p26-25. Genetic polymorphisms in the GHRL gene may affect GHRL production or protein expression, thereby modulating disease risk. Several studies have reported that GHRL polymorphisms might be associated with obesity [25], metabolic syndrome [27], type 2 diabetes [29], breast cancer [30], and colorectal cancer [31].

Only two studies to date have examined the association of GHRL polymorphisms with the risk of liver diseases [17, 26]. The first influence between GHRL rs26312, rs27647, rs26802, rs34911341, rs696217, and rs4684677 polymorphisms and liver fibrosis risk was reported by Moreno et al in 2010 [17]. They contained 284 chronic hepatitis C Spanish patients, and found that GHRL SNPs rs26312 and rs27647 influenced the progression of liver fibrosis in patients with chronic hepatitis C. Another study assessed the relationship of progression of HCC with the GHRL rs27647, rs34911341, and rs696217 polymorphisms in 40 Egyptian patients with chronic hepatitis C, 39 patients with HCV-related HCC, and 40 healthy subjects [26]. The findings of these researchers showed that the GHRL rs34911341 A allele was associated with HCC risk in patients with hepatitis C. Nonetheless, these studies were both performed with a small sample size and only focused on patients with HCV-related diseases. The present study included a relatively large total number of subjects (176 CHB patients, 106 LC patients, 151 HCC patients, and 167 healthy controls) and explored the interaction between GHRL rs26311, rs27647, rs696217 and rs34911341 polymorphisms and risk of HBV-related diseases. A correlation was found between the GHRL rs26311 polymorphism and an increased LC risk. With a larger sample size, our results had a much greater statistical power than did the previous studies.

Our data indicated a gender difference for the degree of impact of the GHRL rs26311 polymorphism on LC risk. Males who carried the rs26311 C allele and the combined GC+CC genotypes in the GHRL gene were at significantly higher risk for LC. Significant differences were not observed in genotype and allele distributions of GHRL rs26311 in females. This gender effect may be due to the possible role of the larger total number of subjects in the male LC group (n = 88) than in the female LC group (n = 18). On the other hand, different types of diets (such as meat and vegetable intake) or lifestyles (such as tobacco and alcohol abuse) or gene–gene and gene–environment interactions between male and female subjects may also play a role.

In this study, we observed that the serum GHRL levels were significantly lower in LC patients compared with the healthy controls. A previous study conducted by Moreno et al. [17] showed that serum GHRL levels decreased in patients with liver advanced fibrosis. Another study performed by Ataseven et al. [39] demonstrated that serum GHRL concentrations were significantly increased in LC patients. Tacke et al. [40] also reported that serum GHRL levels were significantly elevated in Child C LC cases. These inconsistent results may due to no available international standards for the measurement of GHRL, and results from different laboratories and different assays can vary widely, and additionally, ethnic differences in genetic backgrounds may also play a role [20].

Although our study showed positive results, several limitations remain. First, we have no information on known risk factors such as HCV infection, aflatoxin B1 exposure, smoking, and alcohol consumption, so we did not perform the analyses stratified by these risk factors. Secondly, the current study researched only four SNPs in the GHRL gene. It would be interesting to identify more SNPs polymorphisms in the GHRL gene and investigate their association with HBV-related diseases. Finally, participants in our research were recruited from only one hospital in Guangxi and may not be representative of the entire Chinese population. Therefore, further studies are needed to replicate the results in other populations.

In conclusion, our results suggest that the GHRL rs26311 polymorphism is associated with an increased risk of LC in patients with HBV, particularly in men. The serum GHRL levels were significant decreased in LC patients when compared to healthy controls, but no association between rs26311 polymorphism with serum GHRL levels in LC patients was found.

## Supporting Information

S1 Table Association analysis of GHRL polymorphisms between HBV-related patients and healthy controls in males. (DOCX)

S2 Table Association analysis of GHRL polymorphisms between HBV-related patients and healthy controls in females. (DOCX)
