# Supplementary material for: Association of Ghrelin Gene Polymorphisms and Serum Ghrelin Levels with the Risk of Hepatitis B Virus-Related Liver Diseases in a Chinese Population
Source: PLoS One. 2015 Nov 23;10(11):e0143069. doi: 10.1371/journal.pone.0143069 (PMC4658098; doi:10.1371/journal.pone.0143069)
Supplement: S1 Table — (DOCX) [file pone.0143069.s001.docx]

**Association** **of Ghrelin Gene Polymorphisms** **and Serum Ghrelin Levels with the Risk of Hepatitis B Virus-related Liver Diseases in a Chinese Population**

Xiaolian Zhang^1▲^, Limin Zhai^1▲^, Chengzhi Rong^1^, Xue Qin^1^^*^, and Shan Li^1*^

^1^Department of Clinical Laboratory, First Affiliated Hospital of Guangxi Medical University, Nanning, Guangxi, China.

^▲^These authors contributed equally to this work so that they should be considered as the co-first authors.

^*^Corresponding author: Prof. Xue Qin (e-mail: qinxue919@126.com) and Prof. Shan Li (e-mail: lis8858@126.com). Tel: +86-0771-5356052; Fax: +86-0771-865353342.

| **S1 Table.** Association analysis of GHRL polymorphisms between HBV-related patients and healthy controls in males. | | | | | | | | | | | | | |
| --- | --- | --- | --- | --- | --- | --- | --- | --- | --- | --- | --- | --- | --- |
|  | Controls |  | CHB |  |  |  | LC |  |  |  | HCC |  |  |
| SNPs | N=144 (%) |  | N=149 (%) | OR (95% CI) | *P* |  | N=88 (%) | OR (95% CI) | *P* |  | N=131 (%) | OR (95% CI) | *P* value |
| rs26311 |  |  |  |  |  |  |  |  |  |  |  |  |  |
| GG | 53 (36.8) |  | 54 (36.2) | 1 |  |  | 25 (28.4) | 1 |  |  | 56 (42.7) | 1 |  |
| GC | 68 (47.2) |  | 69 (46.3) | 0.991 (0.647-1.518) | 0.968 |  | 44 (50.0) | 1.635 (0.940-2.845) | 0.082 |  | 58 (44.3) | 0.970 (0.611-1.540) | 0.897 |
| CC | 23 (16.0) |  | 26 (17.5) | 1.173 (0.674-2.039) | 0.892 |  | 19 (21.6) | 1.944 (0.999-3.786) | 0.051 |  | 17 (13.0) | 0.657 (0.349-1.238) | 0.194 |
| GC+CC | 91 (63.2) |  | 95 (63.8) | 1.035 (0.690-1.553) | 0.867 |  | 63 (71.6) | **1.729 (1.019-2.933)** | **0.042** |  | 75 (57.3) | 0.887 (0.571-1.377) | 0.593 |
| G alleles | 174 (60.4) |  | 177 (59.4) | 1 |  |  | 94 (53.4) | 1 |  |  | 170 (64.8) | 1 |  |
| C alleles | 114 (39.6) |  | 121 (40.6) | 1.070 (0.810-1.413) | 0.634 |  | 82 (46.6) | **1.416 (1.017-1.972)** | **0.040** |  | 92 (35.1) | 0.849 (0.627-1.150) | 0.290 |
| rs27647 |  |  |  |  |  |  |  |  |  |  |  |  |  |
| TT | 113 (78.5) |  | 118 (79.2) | 1 |  |  | 64 (72.7) | 1 |  |  | 102 (77.9) | 1 |  |
| TC | 30 (20.8) |  | 29 (19.5) | 0.771 (0.521-1.141) | 0.193 |  | 24 (27.3) | 1.216 (0.786-1.881) | 0.380 |  | 29 (22.1) | 0.804 (0.531-1.217) | 0.302 |
| CC | 1 (0.7) |  | 2 (1.3) | 2.211 (0.392-12.487) | 0.369 |  | 0 (0.0) | - | - |  | 0 (0.0) | - | - |
| TC＋CC | 31 (21.5) |  | 31 (20.8) | 0.809 (0.565-1.159) | 0.248 |  | 24 (27.3) | 1.216 (0.786-1.881) | 0.380 |  | 29 (22.1) | 0.804 (0.531-1.217) | 0.302 |
| T alleles | 256 (88.9) |  | 265 (88.9) | 1 |  |  | 152 (86.4) | 1 |  |  | 233 (88.9) | 1 |  |
| C alleles | 32 (11.1) |  | 33 (11.1) | 0.869 (0.594-1.272） | 0.470 |  | 24 (13.6) | 1.097 (0.720-1.673) | 0.667 |  | 29 (11.1) | 0.760 (0.505-1.141) | 0.189 |
| rs696217 |  |  |  |  |  |  |  |  |  |  |  |  |  |
| GG | 97 (67.3) |  | 90 (60.4) | 1 |  |  | 54 (61.4) | 1 |  |  | 91 (69.5) | 1 |  |
| GT | 43 (29.9) |  | 55 (36.9) | 1.408 (0.973-2.039) | 0.070 |  | 31 (35.2) | 1.305 (0.843-2.021) | 0.232 |  | 36 (27.5) | 0.868 (0.579-1.302) | 0.493 |
| TT | 4 (2.8) |  | 4 (2.7) | 1.163 (0.416-3.250) | 0.774 |  | 3 (3.4) | 1.115 (0.360-3.450) | 0.851 |  | 4 (3.0) | 0.734 (0.254-2.125) | 0.569 |
| GT＋TT | 47 (32.7) |  | 59 (39.6) | 0.722 (0.510-1.021) | 0.066 |  | 34 (38.6) | 0.777 (0.516-1.169) | 0.226 |  | 40 (30.5) | 1.172 (0.804-1.708) | 0.409 |
| G alleles | 237 (82.3) |  | 235 (78.9) | 1 |  |  | 139 (79.0) | 1 |  |  | 218 (83.2) | 1 |  |
| T alleles | 51 (17.7) |  | 63 (21.1) | 1.288 (0.935-1.774) | 0.122 |  | 37 (21.0) | 1.202 (0.827-1.746) | 0.335 |  | 44 (16.8) | 0.868 (0.609-1.238) | 0.435 |
| HBV, hepatitis B virus; SNPs, single nucleotide polymorphisms; CHB, chronic hepatitis B; LC, liver cirrhosis; HCC, hepatocellular carcinoma; OR, odds ratio; CI, confidence interval. | | | | | | | | | | | | | |
