# Supplementary material for: Association of Ghrelin Gene Polymorphisms and Serum Ghrelin Levels with the Risk of Hepatitis B Virus-Related Liver Diseases in a Chinese Population
Source: PLoS One. 2015 Nov 23;10(11):e0143069. doi: 10.1371/journal.pone.0143069 (PMC4658098; doi:10.1371/journal.pone.0143069)
Supplement: S2 Table — (DOCX) [file pone.0143069.s002.docx]

**Association** **of Ghrelin Gene Polymorphisms** **and Serum Ghrelin Levels with the Risk of Hepatitis B Virus-related Liver Diseases in a Chinese Population**

Xiaolian Zhang^1▲^, Limin Zhai^1▲^, Chengzhi Rong^1^, Xue Qin^1^^*^, and Shan Li^1*^

^1^Department of Clinical Laboratory, First Affiliated Hospital of Guangxi Medical University, Nanning, Guangxi, China.

^▲^These authors contributed equally to this work so that they should be considered as the co-first authors.

^*^Corresponding author: Prof. Xue Qin (e-mail: qinxue919@126.com) and Prof. Shan Li (e-mail: lis8858@126.com). Tel: +86-0771-5356052; Fax: +86-0771-865353342.

| **S2 Table.** Association analysis of GHRL polymorphisms between HBV-related patients and healthy controls in females. | | | | | | | | | | | | | |
| --- | --- | --- | --- | --- | --- | --- | --- | --- | --- | --- | --- | --- | --- |
|  | Controls |  | CHB | | |  | LC | | |  | HCC | | |
| SNPs | N=23 (%) |  | N=27 (%) | OR (95% CI) | *P* |  | N=18 (%) | OR (95% CI) | *P* |  | N=20 (%) | OR (95% CI) | *P* value |
| rs26311 |  |  |  |  |  |  |  |  |  |  |  |  |  |
| GG | 9 (39.1) |  | 11 (40.7) | 1 |  |  | 4 (22.2) | 1 |  |  | 7 (35.0) | 1 |  |
| GC+CC | 14 (60.9) |  | 16 (59.3) | 0.763 (0.263-2.211) | 0.618 |  | 14 (77.8) | 1.678 (0.530-5.315) | 0.379 |  | 13 (65.0) | 1.193 (0.396-3.591) | 0.753 |
| G alleles | 26 (56.5) |  | 33 (61.1) | 1 |  |  | 17 (47.2) | 1 |  |  | 24 (60.0) | 1 |  |
| C alleles | 20 (43.5) |  | 21 (38.9) | 0.661 (0.313-1.396) | 0.278 |  | 19 (52.8) | 1.063 (0.512-2.207) | 0.870 |  | 16 (40.0) | 0.887 (0.421-1.870) | 0.753 |
| rs27647 |  |  |  |  |  |  |  |  |  |  |  |  |  |
| TT | 19 (82.6) |  | 21 (77.8) | 1 |  |  | 15 (83.3) | 1 |  |  | 17 (85.0) | 1 |  |
| TC+CC | 4 17.4) |  | 6 (22.2) | 1.431 (0.500-4.095) | 0.504 |  | 3 (16.7) | 0.915 (0.306-2.732) | 0.873 |  | 3 (15.0) | 0.871 (0.277-2.745) | 0.814 |
| T alleles | 42 (91.3) |  | 47 (87.0) | 1 |  |  | 33 (91.7) | 1 |  |  | 37 (88.9) | 1 |  |
| C alleles | 4 (8.7) |  | 7 (13.0) | 2.107 (0.800-5.085) | 0.137 |  | 3 (8.3) | 0981 (0.303-3.178) | 0.975 |  | 3 (11.1) | 1,021 (0.309-3.113) | 0.974 |
| rs696217 |  |  |  |  |  |  |  |  |  |  |  |  |  |
| GG | 15 (65.2) |  | 21 (77.8) | 1 |  |  | 11 (61.1) | 1 |  |  | 11 (55.0) | 1 |  |
| GT＋TT | 8 (34.8) |  | 6 (22.2) | 1.534 (0.607-3.836) | 0.366 |  | 7 (38.9) | 0.623 (0.246-1.580) | 0.319 |  | 9 (45.0) | 0.616 (0.242-1.567) | 0.310 |
| G alleles | 37 (80.4) |  | 47 (87.0) | 1 |  |  | 28 (77.8) | 1 |  |  | 28 (7.0) | 1 |  |
| T alleles | 9 (19.6) |  | 7 (13.0) | 0.794 (0.322-1.955) | 0.615 |  | 8 (22.2) | 1.443 (0.637-3.270) | 0.380 |  | 12 (30) | 1.925 (0.863-4.294) | 0.110 |
| HBV, hepatitis B virus; SNPs, single nucleotide polymorphisms; CHB, chronic hepatitis B; LC, liver cirrhosis; HCC, hepatocellular carcinoma; OR, odds ratio; CI, confidence interval. | | | | | | | | | | | | | |
